# Study of the Friction Contact of HIPIMS Magnetron-Sputtered TiB_2_ Against Aluminium at Temperatures up to 300 °C

**DOI:** 10.3390/ma18132975

**Published:** 2025-06-23

**Authors:** Gonzalo G. Fuentes, Marya Baloch, José Fernández Palacio, Pablo Amezqueta, Rebeca Bueno, Jonathan Fernández de Ara, Herbert Gabriel, Cayetano Hernández, Pilar Prieto, Germán Alcalá

**Affiliations:** 1 Asociación de la Industria Navarra—AIN, E31191 Cordovilla, Spain; mbaloch@ain.es (M.B.); jfpalacio@ain.es (J.F.P.); pamezqueta@ain.es (P.A.); jfernandez@ain.es (J.F.d.A.); 2 Plasma Vakuum Technik, Rudolf Diesel Str 7, 64625 Bensheim, Germany; h.gabriel@pvtvacuum.de; 3 Departamiento de Física Aplicada, Instituto Nicolás Cabrera, Universidad Autónoma de Madrid, 28049 Madrid, Spain; cayetano.hernandez@estudiante.uam.es (C.H.); pilar.prieto@uam.es (P.P.); 4 Departamiento de Ingeniería Química y Materiales, Universidad Complutense de Madrid, Ciudad Universitaria s/n, 28040 Madrid, Spain; galcalap@ucm.es

**Keywords:** TiB_2_ coatings, HiPIMS, aluminium adhesion, galling

## Abstract

In this study, we investigated the frictional properties of TiB_2_ films produced by high-power impulse magnetron sputtering and compared them with those of TiN- and CrN-sputtered coatings also made using high-power pulsed discharges. The films were characterised by scanning electron microscopy, Electron Probe Micro-Analysis, nanoindentation and friction tests. Sliding friction analyses were performed against aluminium surfaces at different temperatures, ranging from room temperature to 300 °C. The TiB_2_ coatings exhibited hardness values of about 39 GPa, regardless of the bias potential used between −50 V and −100 V, a low modulus of around 300 GPa and a dense compact columnar microstructure with grain sizes between 51 and 68 nm in diameter. The friction behaviour on aluminium produced the transfer of this element to the films, at rates that depended on the test temperature. The TiN and CrN coatings exhibited low–medium adhesion to aluminium at room temperature and severe transfer during the friction tests at 150 °C. In the case of the TiB_2_ films, the adhesion of aluminium during friction tests was low for temperatures up to 175 °C. In fact, a clear transition of the mild-to-severe adhesion of aluminium on TiB_2_ was observed in the temperature range of 175 °C to 200 °C for the testing conditions evaluated in this study, which was concomitant with the evolution observed for the friction coefficients.

## 1. Introduction

The use of lightweight metallic alloys in the transportation industry, like those based on aluminium, is widespread and constitutes a major drive for the development of energy-efficient vehicles. Aluminium is a soft, light and sticky metal which easily adheres over the tool counter surfaces (i.e., typically highly alloyed tool steel) under applied forces in combination with friction and temperature. These harassing friction conditions severely affect the life span of the tools aimed at manufacturing large series of Al parts, as these are required to be delivered in the vehicle industry. In fact, the combined effect of mechanical load, friction and temperature often triggers the adhesion of Al onto the forming tools, causing production downtimes due to tool maintenance or replacement or defective part finishing, for example built-up edge in machining [1] or galling in hot stamping [2]. In some of the traditional processes to manufacture aluminium parts, the tools are typically preheated to temperatures from 150 °C to 300 °C, while the contact temperatures may rise during the contact friction depending on the type of process and conditions (pressure, speed), cooling performance of the tools, etc.

Vapour deposition coatings constitute a widely extended route to reduce tool wear, and to a lesser extent, prevent the adhesion of sticky working metals [3,4,5,6,7,8,9]. Coating materials such as CrN [5,6], AlTiN [6], diamond-like carbon [7,8,9], or WC-C [7], as well as other surface treatments, have proved that they have good efficiency in reducing the sticking of soft metals, i.e., Al, Mg or stainless steel, on tool materials such as tool steels or hard metals [3,4,5,6,7,8,9]. In this context, the transition metal diborides [10] have also emerged as candidate coatings for this purpose. In particular, titanium diboride (TiB_2_) [11,12] is a paradigmatic coating material due to its excellent thermal stability, high electrical conductivity, high melting temperature, chemical inertness and resistance to mechanical wear.

TiB_2_ coatings can be synthesised using chemical vapour deposition [12], vacuum arc [13], plasma spray [10] and sputtering techniques [14,15,16,17,18,19,20,21,22,23,24], including DC, DC pulsed and, more recently, high-power impulse magnetron sputtering (HiPIMS) [25,26,27]. They belong to the family of hard coatings, as this boride exhibits indentation hardness values well over 25 GPa.

Sputtered TiB_2_ can develop different microstructures, mostly nanocolumnar, depending on the B/Ti molar ratio. Mikula et al. [28] and Lofaj et al. [16], a decade ago, deposited superhard TiB_2_ with indentation hardness values above 30 GPa by DC sputtering, some reaching up to 40 GPa for specific deposition conditions. Fuger et al. [17] reported on the crystalline preferred orientation and mechanical property dependencies over the film stoichiometry for B/Ti molar ratios from 2 up to 3.6. Hellgren et al. [25] investigated the composition and microstructural properties of TiB_2_ deposited by HiPIMS techniques, showing films poor in B due to variations in the sputtered ionisation probabilities for different chemical species. Thörnberg et al. [26] also showed that HiPIMS-type sputtering films of TiB_2_ targets produced under-stoichiometric films with superhardness values up to 43 GPa and an apparent fracture toughness of 4.2 MPa_×_m^1/2^. Finally, there exists an extensive body of literature covering the alloying of TiB_2_ with other light elements such as nitrogen, carbon, silicon [21] or aluminium [29], among others, with the purpose of increasing its toughness and structural strength under high loading conditions, as well as its oxidation resistance at high temperatures.

Along with their high hardness, TiB_2_ coatings are also interesting due to their low adhesion to other metals due to the low chemical activity of their basal planes. Mu et al. [30] reported the benefits of a TiB_2_ overlayer on a micro-stamping Ta-based mould to reduce the adhesion of Al at 450 °C and facilitate the de-moulding processes. Berger et al. [5] already investigated the tribological-driven adhesion of aluminium on sputtered TiB_2_, as well as on commercial TiN, TiAlN and TiCN at ambient temperatures for various friction-normal loads, showing that TiB_2_ outperformed the commercial coatings against the pick-up of Al-counter material. Konca et al. [14] reported the Al transfer during friction experiments on both smooth and rough TiB_2_ films, and compared the results with those on sputtered CrN and cathodic arc-plated TiN, TiAlN and TiCN. The parameters investigated in their work included temperature (ambient and 160 °C), atmosphere exposure (air and Ar-rich) and sliding speed (0.12 m/s, 0.6 m/s). Despite the increased interest in TiB_2_ coatings as anti-sticking interfaces, to the best of our knowledge, there are no studies providing a temperature dependency map of the adhesion rate of Al on HiPIMS-sputtered TiB_2_ coatings.

## 2. Materials and Methods

### 2.1. Coating Deposition

The TiB_2_, TiN and CrN coatings were deposited in a xPro4C industrial system manufactured by Plasma Vakuum Technik GmbH (Bensheim, Germany). The setup chosen for this study was a configuration where 2 rectangular opposing cathodes (700–100 mm of sputtering area) hosted different target compositions depending on the desired coating type. For the deposition of the TiB_2_ coatings, the first cathode hosted a high-purity Ti target (99.2 at.%), which provided the Ti vapour to form the adhesion layer, and the second, a multi-tiled TiB_2_ target with a purity of 99 at.%, which allowed the deposition of TiB_2_ films. For the TiN and the CrN coatings, the two rectangular sources were loaded with Ti and Cr cathodes, respectively, both 99 at.% purity. All the cathodes were fed with an HiPIMS power supply with the model VIESCA HIPIMS V+.

The coatings were deposited on polycrystalline Si wafers and high-speed steel (AISI M2; nominal composition: C 0.8 wt.%, Cr 4 wt.%, Mo 4.5 wt.%, W 6 wt.%, V 2 wt.%, Mn < 0.2 wt.% and Fe-balance) which was quenched-tempered, with a hardness of 62 HRC. The temperature within the reactor chamber was maintained in the range of 400–450 °C along the whole deposition process. The samples were positioned in a biassed holder with a two-fold rotation, so that their minimum distance to the cathode surfaces was 9–10 cm. The steel specimens were mirror-polished to Ra values of around 15 nm. All the coatings exhibited good adhesion strength on the M2 substrates, with HF values ranking between 1 and 2 in the Daimler-Benz Rockwell C indentation adhesion test, where HF1 was excellent and HF6 was the poorest adhesion [31].

**Figure 1:**
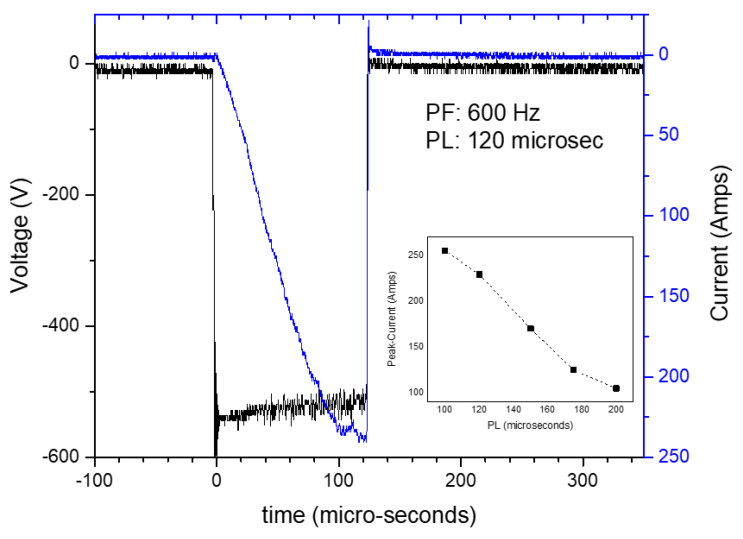
Evolution of the HiPIMS peak current of the TiB_2_ cathode as a function of the pulse length for a constant pulse frequency, and V-I pulse shape for the selected pulse frequency.

The film deposition consisted of a series of sequential steps covering the reactor heating, target cleaning, substrate Ar^+^ etching, deposition of an adhesion layer and finally the TiB_2_, TiN or CrN coating deposition using the HIPIMS mode. The primary parameters for the 3-step sequence (excl. the target cleaning) are shown in Table 1 for the three coatings. The specimen TiB_2_/50 was deposited using a bias potential of −50 V, similar to the bias used in previous reference studies for TiB2 coatings by HIPIMS: −50 V [17] or −60 V [29]. A second TiB2 specimen, TiB_2_/100, was deposited using a slightly higher bias of −100 V, to monitor possible changes in hardness or surface finishing properties. The bias voltage for the TiN and CrN coatings was kept to −100 V. The HiPIMS power, pulse frequency and pulse length on the TiB_2_ target were set to 5 kW, 600 Hz and 120 μs respectively. These conditions allowed us to obtain the maximal possible peak currents during the HIPIMS mode, while reducing the production of arcs. More specifically, Figure 1 shows the voltage and current pulse shape for the final selected pulse length (120 microsec) and the peak current dependencies on the pulse length. The pulse frequency and length chosen provided a good trade-off between deposition speed and a moderate level of arching. The values selected for the TiN deposition were 5 kW, 1000 Hz and 100 μs, and in the case of CrN, they were 5 kW, 750 Hz and 100 μs.

### 2.2. Coating Characterisation

The film outer surfaces were analysed by Electron Probe Micro-Analysis (EPMA) using a JXA-IHP200F microprobe available at the Spanish National Centre for Electron Microscopy (UCM) (Madrid, Spain). The system is capable of accurately detecting and quantifying light elements from Be, and it is equipped with a spectrometer for energy-dispersive X-ray and another for Soft X-ray Emission.

The coating microstructures in the top-view and cross-section were characterised using a field emission scanning electron microscope (SEM) HITACHI S-4800 (Tokyo, Japan), equipped with a secondary electron detector and an electron dispersion spectrometer (EDS). The top-view images were used to measure the grain sizes of the specimens using the intersect method of the ASTM E112-13 (2021) Standard Test Methods for Determining Average Grain Size [32].

The indentation hardnesses (H) and reduced Young moduli of the coatings were determined by nanoindentation using a NanoIndenter XP (MTS) with a Berkovich diamond tip with an elastic modulus of 1140 GPa and a Poisson’s ratio of 0.07. The tip geometry was calibrated on a fused silica sample using the Oliver and Pharr method [33]. The measurements of H and E’ as a function of the indentation depth were carried out using the Continuous Stiffness Measurement (CSM) operation mode, allowing an easy dismissal of measurements with substrate effects, starting at about 10% of the film’s thickness, according to the literature [34]. Thermal drift rate was measured before each test to correct the tip displacement into the samples, making sure it was always below 0.08 nm s^−1^. The indentations were carried out at a constant loading rate of 0.05 N/s up to a maximum load of 10 mN. The conclusions achieved were the outcome of standard statistical analyses over the 20 measurements performed on each sample.

The coefficient of friction (COF) evolutions on the coating outer surfaces were tested against 6 mm diameter aluminium balls (pure Al with some Fe traces), mounted in a ball-on-disc configuration using an applied normal load of 5 N, and a rotation radius and linear sliding speed of 11 mm and 10 cm/s, respectively. These tests were conducted on the TiB_2_ at room temperature (RT), 150 °C, 175 °C, 200 °C, 250 °C and 300 °C; and at RT and 150 °C on TiN and CrN for comparison. This selection obeys the result of a literature search on the temperature ranges recorded in conventional operations, such as machining, hot stamping or high-pressure die casting of aluminium parts. In machining for example, Khedir et al. [35] calculated the drilling tool surface temperature to be from 160 °C up to 360 °C depending on the feed rate and preheating conditions; while in a recent paper, Lui et al. [36] reported a comprehensive literature survey of typical testing temperature conditions for aluminium hot-stamping moulds. These temperature ranges were from ambient up to 400 °C, with interims of 150, 250, 300 °C, etc.

Each test was repeated 3 times, and the friction paths were subsequently inspected after 1500 cycles using the backscattered electron detector of the SEM instrument. These analyses allowed us to qualitatively identify the surface coverage of aluminium transferred from the ball to the coatings during sliding as a function of the different testing temperatures.

### 2.3. Thermodynamic Simulations (CALPHAD Method)

The chemical interaction between the materials under the friction tests has also been studied from the thermodynamic point of view. The commercial software Thermo-Calc 2020b was used to find stable phases that can be formed in thermodynamic equilibrium during the friction tests. The relative chemical activity among the elements present in the interface is an important parameter affecting Al sticking to the tool material and thus its friction behaviour [7,8]. Simulations were performed at different temperatures and pressures to evaluate the influence of the chemical nature of the coatings under the experimental conditions described in the friction test section of this work.

## 3. Results and Discussion

### 3.1. Composition and Microstructure

Table 2 shows the thicknesses, average grain sizes, statistical roughness Ra and Rq, indentation hardnesses and reduced moduli of the coatings. The TiB_2_ coatings reached average thicknesses of 1.4–1.5 μm, while 0.75 μm was reached for the TiN and 1.4 μm in the case of the CrN.

The compositions of the TiB_2_ films, as measured by EPMA, indicated a boron-to-titanium atomic ratio of around 2.1, with an error margin of 0.1, which is similar to that expected from the nominal composition of the target (B/Ti = 2). The composition of the TiN and CrN films was stoichiometric for the CrN and slightly sub-stoichiometric in the case of the TiN.

The B/Ti atomic ratio for sputtered TiB_2_ coatings has received the attention of the scientific community over the last decade. For example, Hellgren et al. [25] reported B/Ti atomic ratios of around 1.4 and 1.5 due to a higher ionisation rate of Ti over B during the HiPIMS process. Thörnberg et al. [26] found B/Ti at ratios ranging from sub-stoichiometric B/Ti = 1.42 to stoichiometric B/Ti = 2. Other authors have reported opposite trends, for example, Nedfors et al. [37] found over-stoichiometric B/Ti at ratios of up to 2.5 for HiPIMS-deposited films, which was attributed to the specific combination of various experimental effects, such as chamber pressure as well as target-to-substrate distance and angle.

**Figure 2:**
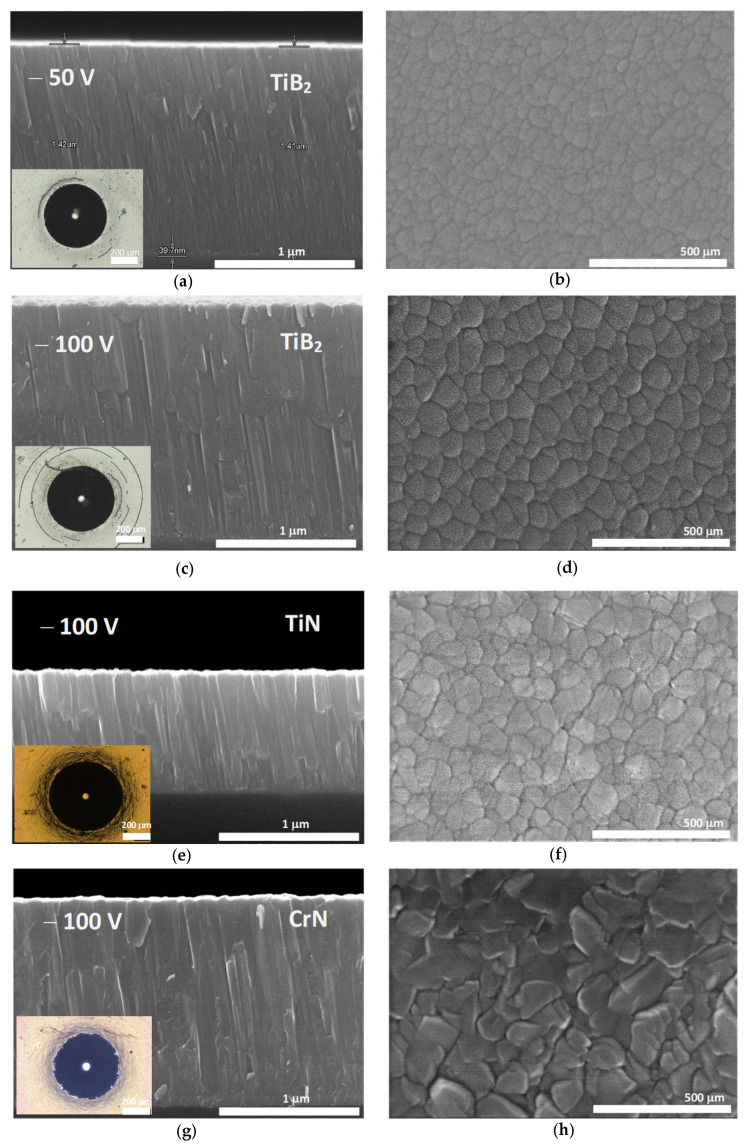
SEM micrographs of the TiB_2_ films deposited by HiPIMS using different BIAS voltages. Cross-sectional micrograph with the Rockwell hardness test image has been shown in (**a**) TiB_2_ @ −50 V, (**c**) TiB_2_ @ −100 V, (**e**) TiN @ −100 V, (**g**) CrN @ −100 V. Surface morphology is shown in (**b**) TiB_2_ @ −50 V, (**d**) TiB_2_ @ −100 V, (**f**) TiN @ −100 V, (**h**) CrN @ −100 V.

Figure 2 shows the coating microstructures, in the cross-section (left) and top-view (right), as observed by SEM using secondary electrons. These micrographs aim to highlight film topographies and their columnar nanostructures, where we can observe that all the coatings exhibited compact and dense cross-sectional nanostructures formed by columns. This growth mode is a characteristic feature of the HiPIMS processes [38] due to the high ion-to-neutrals ratio within the sputtering plasma.

The top-view images reveal a low dispersion of grain morphology and size structure. The specimens TiB_2_/50 and TiB_2_/100 show (cf. Table 2) an average grain size of about 51 nm and 68 nm, respectively. The slightly bigger size displayed by the TiB_2_ specimen deposited under the largest bias potential suggests the greater mobility of the arriving atoms as the bias increases, which allows for the faster kinetic growth of the columnar nuclei. In addition, the grain sizes of the TiN and CrN specimens are, respectively, 62 nm and 78 nm (cf. Table 2), similar to those of the TiB_2_ films. In all cases, the top-view images also reveal the high compactness of the developed nanocolumnar grains, in agreement with the observation of the film cross-sections.

The imprints after the Rockwell adhesion tests on the M2 substrates are shown in the bottom-left in sets in Figure 2, showing, for all the specimens, a qualification of high adhesion strength. The brittle circular cracks observed in the biassed films do not respond to adhesive failures but to crack initiation and propagation in the TiB_2_ films when the steel substrate surface is heavily strained by the Rockwell indenter.

The surface roughness of all the coatings measured over the steel barely increases with respect to that of the as-polished substrates. The TiB_2_/50 specimen shows Ra/Rq values of 28.2/45.0 nm while the measured roughness outcome is 35.4/62.8 nm in the case of the TiB_2_/100. The TiN specimen shows Ra and Rq of 33.5 nm and 58.7 nm, and the CrN specimen yields 16.9 nm and 39.8 nm, respectively.

### 3.2. Indentation Hardness and Modulus

Table 2 presents the values of the indentation hardnesses H and moduli E’ of the coatings deposited on the M2 steel substrates. The hardnesses H of the TiB_2_ specimens exhibit uniform values around 39 GPa regardless of the bias potential applied during the deposition. The indentation moduli also display uniform values of about 300 GPa regardless of the bias potential applied. The hardness values of the TiN and CrN specimens deposited by HiPIMS are 27.1 GPa and 21.2 GPa, respectively.

The values of H for the biassed TiB_2_ specimens approach those of superhardness (H values of 39 GPa close to the 40 GPa threshold) and are in concordance with various references in the literature [17,25,26,37]. For example, Fuger et al. [17] reported that superhardness (i.e., H > 40 GPa) in TiB_2_ is driven by a preferred {0001} orientation of its crystallographic lattice, while film hardnesses decrease significantly for $101\bar{1}$ and 1000-oriented films. Thörnberg et al. [26] attributed the superhardness of TiB1.43 films deposited by HiPIMS to a combined effect of low grain size, larger densification and a high concentration of Ti-rich stacking faults; all these effects act as barriers for dislocation slip. Additionally, superhard TiB_2_+z coatings with excess B have also been obtained by magnetron sputtering [37], achieving this high hardness due to the formation of B bulk interstitials combined with the formation of B-rich surfaces around the TiB_2_ nanograins. This effect was also supported by density functional theory (DFT) calculations [39]. On the other hand, the values of the nanoindentation moduli are smaller than those measured at [26], i.e., 511 GPa for TiB1.43, and at [33], i.e., 450–480 GPa. This suggests that the coatings produced in this work exhibit a good resilience level (elastic energy absorption capacity before yielding).

### 3.3. Friction Against Aluminium as a Function of Temperature

**Figure 3:**
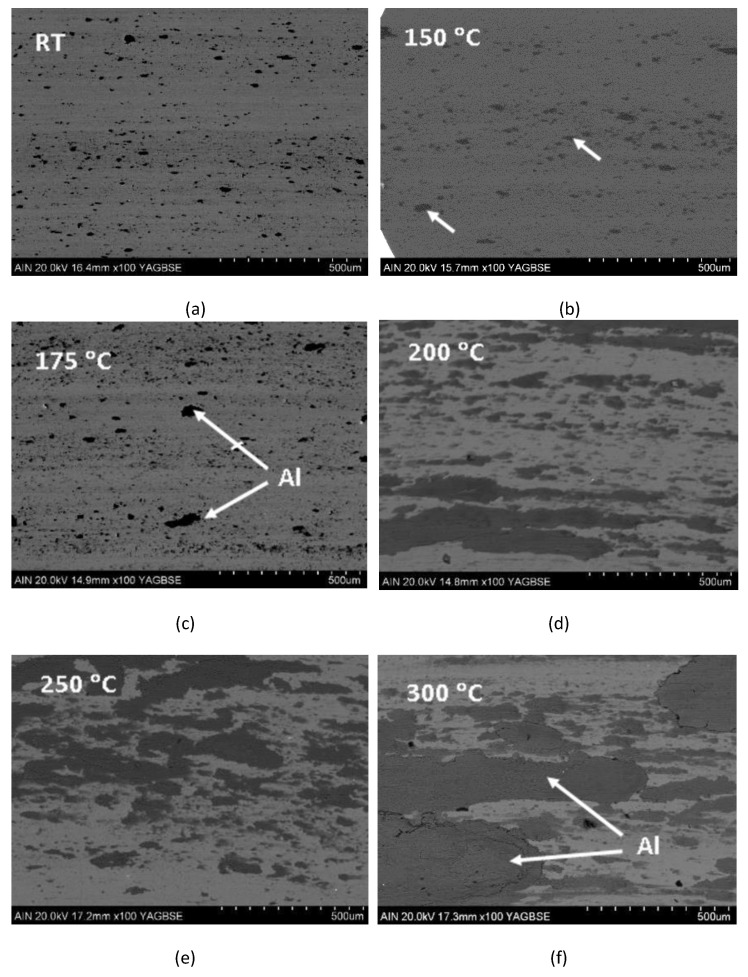
SEM images of the friction path between the TiB_2_/50 surfaces and the aluminium counter ball after the sliding tests at (**a**) RT, (**b**) 150 °C, (**c**) 175 °C, (**d**) 200 °C, (**e**) 250 °C and (**f**) 300 °C.

To study the friction of the TiB_2_ coating surfaces against aluminium and its temperature dependencies, we have only presented the results of the TiB_2_/50 specimen, since the bias of −50 V, or very similar (i.e., −60 V), has been applied in recent papers reporting on TiB_2_ coatings deposited in the HIPIMS mode [17,29,37]. In fact, the results obtained for the TiB_2_/100 specimen were qualitatively similar to those of the TiB_2_/50 specimen, which was expected since their composition, hardness and microstructure were also similar. Figure 3 shows the SEM micrographs, in backscattered mode, of representative portions of friction paths on the TiB_2_/50 specimen after 1500 sliding cycles of the aluminium counter balls at room temperature, 150 °C, 175 °C, 200 °C, 250 °C and 300 °C, as labelled.

The friction path on the TiB_2_/50 specimen tested at ambient temperature shows a uniform unworn surface (grey background) and the presence of small dark grey spots (<50 nm) corresponding to aluminium transferred from the ball to the coating. The fact that the TiB_2_ does not show hints of wear is an expected result, due to the difference in hardness between the two materials, and as this is in agreement with previous results reporting on the friction tests of TiB_2_ coatings sliding on aluminium alloys AA7075 at room temperature [40]. The friction paths of the specimens tested at 150 °C and 175 °C reveal a similar qualitative appearance compared to the one assessed at ambient temperature, but with about 20–30% larger Al spots on the friction path produced at 175 °C (white arrows). At 200 °C, the wear of the TiB_2_ is still negligible, though a much larger presence of Al transferred to the coating surface is noticed. This Al transfer becomes very large at temperatures from 200 °C and above (notice that 200 °C corresponds to about 30% of the aluminium melting temperature Tmax 660 °C). At 300 °C (45% of the Tm of aluminium), the coverage index of the TiB_2_ surfaces is larger than 60% (white arrows pointing to the aluminium transferred). No significant changes are expected in the TiB_2_ surface chemistry due to air annealing, according to the results from Dorri et al. [41], who reported very low oxidation kinetics at such a temperature in air.

**Figure 4:**
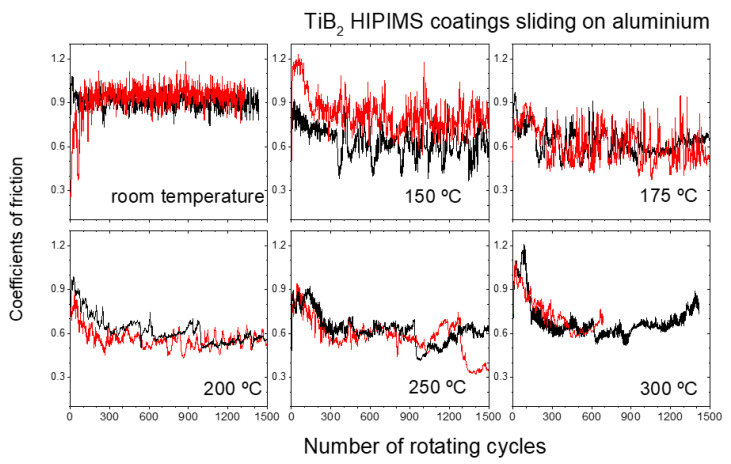
Coefficient of friction evolution for the sliding tests performed on TiB_2_/50 at room temperature, 150 °C, 175 °C, 200 °C, 250 °C and 300 °C, as labelled. The black and red curves correspond to repeated tests.

Figure 4 depicts the resulting evolution of the coefficients of friction (COFs) between the TiB_2_ films and the Al counter balls measured at room temperature, 150 °C, 175 °C, 250 °C and 300 °C, as labelled. Two repeated COF evolutions have been added per temperature in order to illustrate the repeatability of the results. The COFs measured over the TiB_2_/100 specimen (deposited using a bias voltage of 100 V negative) exhibited the same qualitative behaviour (the COFs of TiB_2_/50 and TiB_2_/100 are presented together in the supplementary content as Figure S1). The COFs of the TiB_2_-Al system at room temperature unveil a uniform consistent evolution with values around 0.9. No frictional effects such as stick-slip or the formation of tribofilms can be inferred along the tests. However, the behaviour at 150 °C reveals an overall progressive decrease in the COF from 0.9 down to 0.6. In this case, noticeable low frequency fluctuations of the COF are observed, which could be attributed to stick-slip phenomena of Al partially transferred to the TiB_2_ surface and loosened after a few cycles. This is manifested by larger peak amplitude in the COF vs. sliding cycle graphs. This interpretation is consistent with the resulting friction path left after the analysis, which reveals some traces of Al residues over the TiB_2_ surface. The COF evolution of the specimen tested at 175 °C exhibits similar behaviour as that of the sample tested at 150 °C, the only remarkable difference being that the low frequency fluctuations due to the Al recurrent stick-slip and detachment process starts at the beginning of the test, while at 150 °C this process starts at about 350 cycles. Up to this temperature all the COF curves show a noisy appearance, denoted by a low amplitude and high-frequency oscillations. At 250 °C and 300 °C the COFs also evolve from 0.9 to 0.6 for the first 1300 cycles. From this moment the COF suffers a significant drop to values of about 0.35, probably due to the formation of an Al tribofilm on the coating, making the probe slide from that moment on a TiB_2_ + aluminium surface; this observation is consistent with the SEM micrograph of the corresponding frictional path (cf. Figure 3).

The observed results indicate the existence of an onset temperature above which the transfer rate of aluminium to the TiB_2_ surface increases. For the experimental conditions of this study, such onsets are located within the range between 175 °C and 200 °C, according to the SEM analyses presented in Figure 3. It is noteworthy that the mentioned onset is close to the limit of 0.3 Tm (where T_m_ is the nominal Al melting temperature). At this onset, creep effects (i.e., plastic flow at loads below the material’s yield strength σ_y_) increase significantly, which would produce a damping effect, reducing low-frequency noise in the curve and favouring the subsequent formation of an aluminium tribofilm over the TiB_2_ coating after 1300 cycles. The tribofilm presence in this second stage effectively reduces the COF down to values around 0.35, as determined by the friction test at 250 °C.

On the other hand, the chemical nature of the coating is well known to play a major role in the sticking phenomena during counter material friction. In fact, previous studies have already reported on the temperature transition thresholds between the mild and severe adhesion of aluminium onto diverse coatings during friction experiments. For example, previous works of the authors have reported [7,8] that the friction evolution at an interface between sputtered WC-C and Al with 99% purity exhibited a mild–severe Al adhesion transition onset, reflected as a sudden increase in the COFs from still values of about 0.35 to noisy fluctuating values of about 0.85, with the manifestation of this behavioural change being strongly temperature dependent, and taking place at a smaller number of cycles when the test is performed at a higher temperature. This type of transition has been investigated by Bolvardi et al. [42,43] for Mo2BC surfaces and aluminium by means of ab initio and molecular dynamics techniques, showing that an onset of Al transfer is caused by the formation of Al-MoC bonds through Al-O bridges [43].

**Figure 5:**
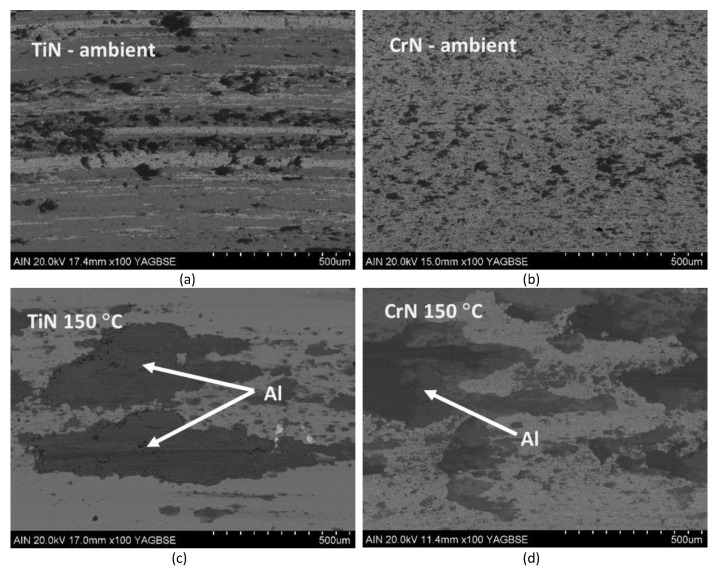
SEM images of the friction path between the TiN and CrN surfaces and the aluminium counter ball after the sliding tests at (**a**,**b**): room temperature and (**c**,**d**): 150 °C.

**Figure 6:**
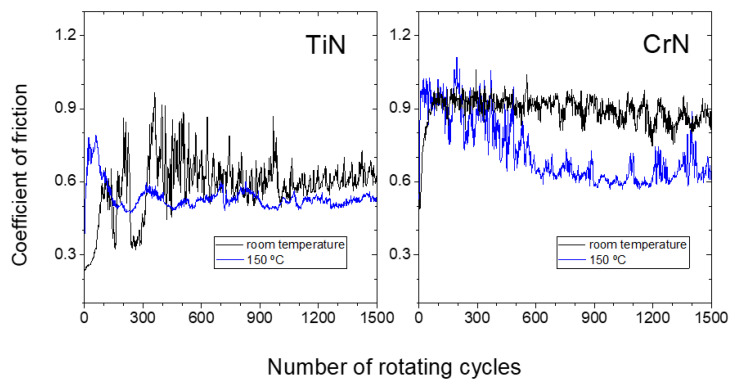
Coefficient of friction evolution for the sliding tests performed over the TiN and CrN coatings at room temperature (black lines) and 150 °C (blue line).

We have carried out the same frictional tests at RT and 150 °C on the deposited TiN and CrN films. Figure 5 shows the SEM images in backscattered mode over the paths produced by aluminium after sliding on TiN and CrN under identical testing conditions to those used on the TiB_2_ films. In addition, Figure 6 shows the evolution of the COFs for each of the tests conducted. Figure 5 provides evidence of the influence of temperature over the aluminium transfer onto the TiN and the CrN film surfaces. At ambient temperature, the amount of aluminium transferred to the TiN coatings is slightly larger than that observed for TiB_2_. In fact, the image of the track over TiN shows some wear damage scars along the sliding direction. The sliding path at room temperature on CrN still shows a coverage of aluminium bigger than that observed for the TiB_2_. On the other hand, no further surface damage is observed on CrN. At 150 °C, the transfer of aluminium onto the TiN and the CrN surfaces is much larger with respect to that monitored at room temperature, and significantly larger than that observed on the TiB_2_ specimens at 150 °C.

The COFs of the TiN specimens, as shown in Figure 6, reflect the findings revealed by the micrographs in Figure 5 well. At room temperature, the COF of TiN shows variable values, which is an indication of changes in the sliding interface, likely due to the adhesion and detachment of aluminium together with the important influence of wear damaging effects, until a more stable COF of around 0.6 is reached subsequent to ~400 cycles. At 150 °C a COF of about 0.55 is reached after ~120 sliding cycles, indicating the formation of a stable tribological interface. Both the ductility and creep properties of the abundant aluminium present in the interface at this temperature play a key role to reduce the COFs high-frequency fluctuations in comparison to the tests at room temperature, producing a damping effect in the recorded data.

The COFs measured on the CrN specimens at room temperature remain constant at values around 0.9 after ~100 cycles (cf. Figure 6), reflecting a stable sliding interface due to the absence of wear. At 150 °C, the COF evolves from 0.9 down to values around 0.6, similar to that on the TiN and the TiB_2_. If we look at the three friction graphs on the three different coatings at 150 °C (Figure 4 and Figure 6), and if we take into account the damping effect of the Al stuck to the interface on the COF noise fluctuations, we can deduce that the amount of Al steadily stuck on the coatings is larger on the TiN that the CrN, and larger on the CrN than the TiB_2_. These results agree with the SEM observations shown in Figure 3 and Figure 5 at 150 °C. It is worth noting that the backscattering micrographs on the CrN sliding track unveil diverse contrast regions, with dark areas where the Al tribofilm is well formed, light areas without Al and wide areas of intermediate contrasts where the Al tribofilm is within the process of formation, and the detachment and sticking of Al is still taking place and leading to a more noisy COF curve compared to the TiN surface.

Additionally, the slightly lower COF measured on the TiN in the steady region of the graph (0.55) may also indicate larger amounts of stable Al on the film surface. However, the average value measured for CrN and TiB_2_ at this temperature, ~0.6 in both cases, indicates that at this intermediate stage of tribofilm formation, the amount of stable Al in the interface is not the only parameter affecting the COF mean value, although it is a key factor in its fluctuations along this stage of the tests. Nevertheless, comparing all the experimental results presented in this work, we can conclude that after 1500 cycles of the friction test, the tribofilm formation is at an earlier stage under the same experimental conditions for TiB_2_, compared to both CrN and TiN.

The thermodynamic simulations performed show a negligible temperature and pressure dependence on the experimental range for these parameters in this work. Nevertheless, the theoretical analysis unveils a high chemical affinity between Al and N, in comparison to that between Al and B. The electronegativity difference between the elements in the first pair is more than 300% larger compared to the second pair. This explains that in thermodynamic equilibrium, the simulation on TiN and CrN leads to the formation of an AlN wurtzite phase, which requires the degradation of the original coating nitrides (Table 3). Although in the experimental conditions in this work, our system is still far from thermodynamic equilibrium, and AlN has not been experimentally detected, the chemical affinity between Al and N is likely to play a key role in assisting Al in sticking to the nitride films. Contrarily, the simulation of the TiB_2_ film reflects that, for the experimental conditions investigated, the formation of AlB_2_ does not take place, maintaining the original TiB_2_ phase unaltered. In the case of not having enough Al in the interface and even at high temperatures, metallic Al is more stable than AlB_2_, compelling the formation of this compound.

These theoretical outcomes support the experimental findings presented here and altogether highlight the overperformance of the TiB_2_ film surfaces in preventing the adhesion of aluminium, in comparison to TiN and CrN films with similar microstructures. The results agree with the previous findings reported in the literature. For example, Berger et al. [40] found that low-residual-stress, TiB_2_-sputtered films exhibited a lower tendency to adhere aluminium at room temperature than TiN on a block rotational configuration in which the coated surface is always in contact with fresh testing material. Konca et al. [14] showed that the aluminium coverage over sputtered TiB_2_ films after friction tests at room temperature was also smaller than that of sputtered CrN and arc-plated TiN, with all coatings having similar values of hardness and surface roughness, in agreement with the results in this work. At testing temperatures of 160 °C, Konca et al. [14] did not observe any significant difference on the aluminium transferred to TiB_2_ with respect to that measured at room temperature, regardless of the surface finishing of the samples. On the contrary, they observed a decrease in transfer at 160 °C on bare M2 steel, arc-deposited TiN and TiCN films, which seems to contradict the results observed in this study.

## 4. Conclusions

In summary, in this study, we have tested the hardness, roughness and microstructure of TiB_2_ coatings deposited using HIPIMS methods. The HIPIMS deposition parameters led to compact, well adhered TiB_2_ coatings with hardness values of 39 GPa regardless of the bias potential used, which nearly qualified the coatings within the superhardness group of films.

Concerning the friction tests, it has been shown that a quantitative temperature threshold for the aluminium adhesion to TiB_2_ in the range of 175–200 °C, under the testing parameters used in this study, clearly outperforms the adhesion properties of benchmark coatings such as TiN and CrN also deposited using HIPIMS techniques.

A damping effect on the noise of the COF curves has been observed at testing temperatures where creep and ductility become apparent in aluminium. This outcome is dependent on the amount of aluminium stably stuck on the coating, becoming a secondary way to compare the stage of evolution of the aluminium tribofilm formation on different surfaces.

The chemical affinity of Al for the elements in the investigated films, by means of thermodynamic simulations using the CALPHAD method, predicts better results of the boride coatings compared to nitride coatings, which supports the present experimental findings.

## Figures and Tables

**Table 1:** Deposition parameters for the coating batches: TiB_2_, CrN and TiN.

	TiB_2_	CrN	TiN
**Heating**			
Temperature	400	400	400
Time	60 min	60 min	60 min
**Ar^+^ Ion Etching**			
Substrate Bias	−300 V	−300 V	−300 V
Cr-HiPIMS Power	0.7 kW	0.7 kW	0.7 kW
Time	15 min	15 min	15 min
**Cladding Layer**			
Sputter Mode/Target	HiPIMS/Ti	HiPIMS/Cr	HiPIMS/Ti
Substrate Bias (V)	−100 V	−100 V	−100 V
HiPIMS Sputtering Power	5 kW	5 kW	5 kW
**Deposition**			
Sputter Mode/Target	HiPIMS/TiB_2_	HiPIMS/Cr	HiPIMS/Ti
Sputtering Power	5 kW	5 kW	5 kW
Pulse Frequency	600 Hz	750 Hz	1000 Hz
Pulse Length	120 μs	100 μs	100 μs
Substrate DC Bias	−50/−100 V	−50 V	−100 V
Ar Gas Flow	120 sccm	90 sccm	90 sccm
N_2_ Gas Flow	-	75 sccm	30 sccm
Temperature	400–450 °C	400–450 °C	400–450 °C
Deposition Time	2 h	2 h	4 h

**Table 2:** Composition, thickness, grain size, roughness and indentation hardness and modulus of the HiPIMS-sputtered specimens.

Sample	Composition	Thickness (μm)	Grain Size (nm)	Ra (nm)	Rq (nm)	H (GPa)	E’ (GPa)
TiB_2_/50	TiB2.1	1.4	51 ± 6	28.2 ± 1.8	45.0 ± 2.0	39.8 ± 2.1	310 ± 5
TiB_2_/100	TiB2.1	1.5	68 ± 6	35.4 ± 2.1	62.8 ± 1.9	39.5 ± 3.1	290 ± 10
TiN	TiN0.8	0.75	62 ± 6	33.5 ± 1.7	58.7 ± 3.8	27.1 ± 1.8	301 ± 13
CrN	CrN	1.4	78 ± 7	16.9 ± 1.7	39.8 ± 5.0	21.2 ± 2.3	243 ± 16

**Table 3:** CALPHAD simulations for the three coatings in the presence of metallic Al. The temperature and pressure range of validity of these results are from 25 to 300 °C and from 1 to 10 atm, respectively. The outputs are the stable phases in thermodynamic equilibrium under the experimental conditions of this work. * (x = 0.23).

Simulation 1			
Input (mol fraction)		Output (mol fraction)	
Al	0.50	0.44	Al
TiN	0.50	0.44	TiN_1−x_ *
		0.12	AlN
**Simulation 2**			
Input (mol fraction)		Output (mol fraction)	
Al	0.50	0.50	CrAl
CrN	0.50	0.50	AlN
**Simulation 3**			
Input (mol fraction)		Output (mol fraction)	
Al	0.50	0.50	Al
TiB_2_	0.50	0.50	TiB_2_

## Data Availability

The original contributions presented in this study are included in the article/Supplementary Material. Further inquiries can be directed to the corresponding author.

## Supplementary Materials

The following supporting information can be downloaded at: https://www.mdpi.com/article/10.3390/ma18132975/s1, Figure S1: Comparison of the coefficient of friction evolution for the sliding tests performed on TiB2/50 and TiB2/100 at room temperature, 150 °C, 175 °C, 200 °C, 250 °C and 300 °C.

## Abbreviations

The following abbreviations are used in this manuscript:

TiB_2_	Titanium Diboride
*TiN*	Titanium Nitride
*CrN*	Chromium Nitride
*TiCN*	Titanium Carbonitride
*TiAlN*	Titanium Aluminium Nitride
*TiN*	Titanium Nitride
Al-MoC	Aluminium–Molybdenum Carbide
AlB_2_	Aluminium Diboride
WC-C	Tungsten Carbide–Cobalt
AlTiN	Aluminium Titanium Nitride
DLC	Diamond-Like Carbon
Al	Aluminium
Mg	Magnesium
B/Ti	Boron/Titanium
*Ti*	Titanium
*Cr*	Chromium
*Fe*	Iron
*Ar*	Argon
*HiPIMS*	High-Power Impulse Magnetron Sputtering
DC sputtering	Direct Current Sputtering
V	Voltage
µm	Micrometre
nm	Nanometre
GPa	Giga Pascals
MPa_×_m^1/2^	Megapascal Metre to the Power of One-Half
m/s	Metres Per Second
at.%	Atomic Percent
mm	Millimetre
nm	Nanometre
RT	Room Temperature
°C	Celsius
Si	Silicon
HRC	Rockwell C Hardness
HF	Enthalpy of Formation
μ	Micro
Hz	Hertz
kW	Kilo Watt
Amps	Amperes
N/s	Newton Per Second
mN	Mili Newton
cm/s	Centimetres Per Second
Ra	Roughness Average
Rq	Root Mean Square Roughness
EPMA	Electron Probe Micro-Analyzer
