# Supplementary material for: Study of the Friction Contact of HIPIMS Magnetron-Sputtered TiB2 Against Aluminium at Temperatures up to 300 °C
Source: Materials (Basel). 2025 Jun 23;18(13):2975. doi: 10.3390/ma18132975 (PMC12250657; doi:10.3390/ma18132975)
Supplement: Supplementary file 1 [file materials-18-02975-s001.zip › materials-3619699-supplementary.pdf]

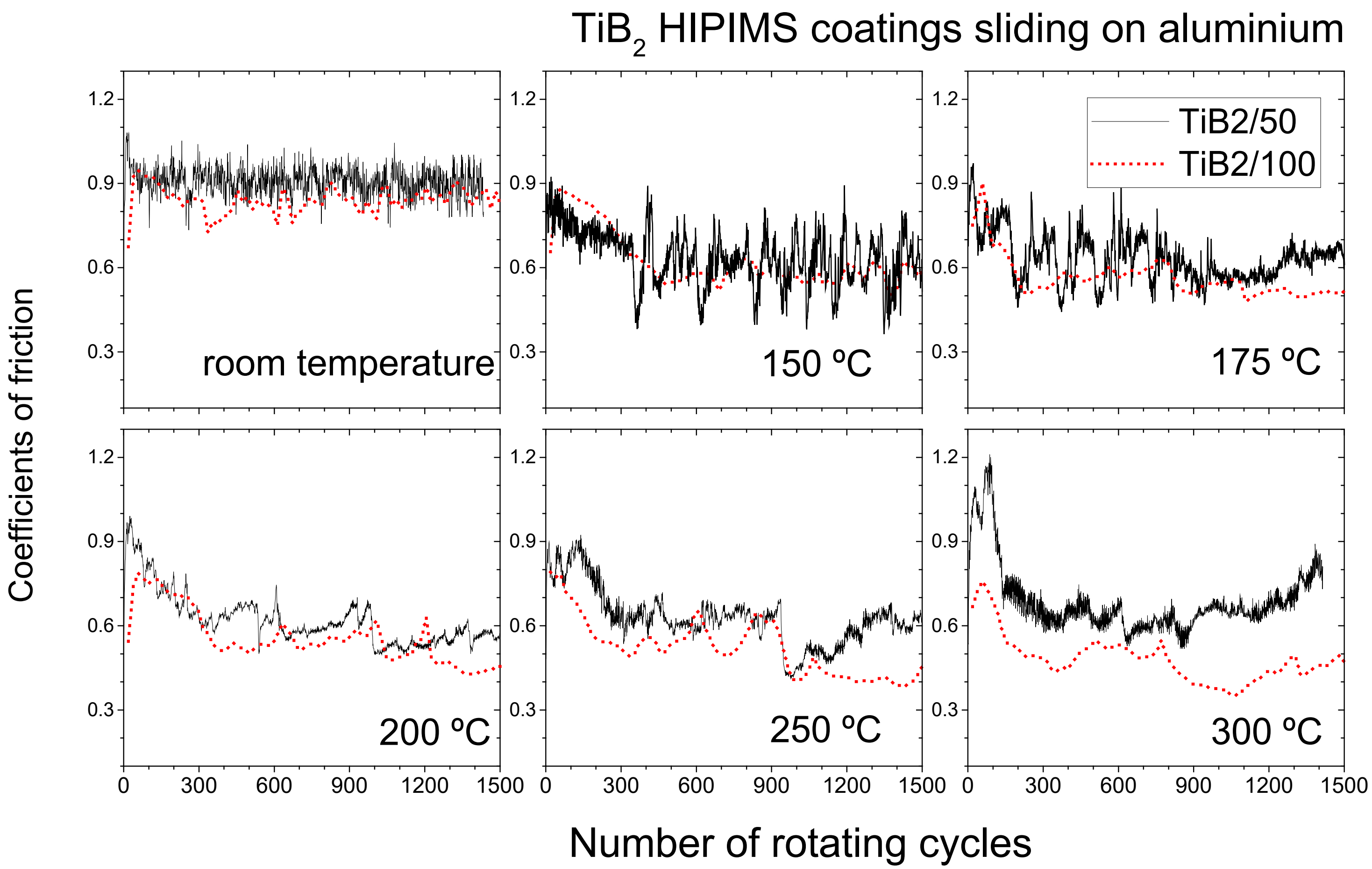

Figure S1. Comparison of the coefficient of friction evolution for the sliding tests performed on TiB_2/50 and TiB_2/100 at room temperature, 150 °C, 175 °C, 200 °C, 250 °C and 300 °C.
